# Inhibition of *Toxoplasma gondii* Growth by Dihydroquinine and Its Mechanisms of Action

**DOI:** 10.3389/fcimb.2022.852889

**Published:** 2022-05-11

**Authors:** Aarin M. Huffman, Joseph A. Ayariga, Audrey Napier, Boakai K. Robertson, Daniel A. Abugri

**Affiliations:** ^1^ Department of Biology, College of Arts and Sciences, Tuskegee University, Tuskegee, AL, United States; ^2^ Department of Biological Sciences, Alabama State University, Montgomery, AL, United States; ^3^ Biomedical Engineering Program, Alabama State University, Montgomery, AL, United States; ^4^ Microbiology PhD Program, College of Science, Technology, Engineering and Mathematics, Montgomery, AL, United States; ^5^ Laboratory of Ethnomedicine, Parasitology, and Drug Discovery, College of Science, Technology, Engineering and Mathematics, Montgomery, AL, United States

**Keywords:** dihydroquinine, inhibition, ROS, mitochondria, infectivity, *T. gondii*

## Abstract

*Toxoplasma gondii* is a zoonotic parasite that infects the brain of humans and causes cerebral toxoplasmosis. The recommended drugs for the treatment or prophylaxis of toxoplasmosis are pyrimethamine (PY) and sulfadiazine (SZ), which have serious side effects. Other drugs available for toxoplasmosis are poorly tolerated. Dihydroquinine (DHQ) is a compound closely related to quinine-based drugs that have been shown to inhibit *Plasmodium falciparum* and *Plasmodium berghei* in addition to its anti-arrhythmia properties. However, little is known about the effect of DHQ in *T. gondii* growth and its mechanism of action *in vitro*. In this study, we report the anti-*Toxoplasma* and anti-invasion properties of DHQ. DHQ significantly inhibited *T. gondii* tachyzoite growth with IC_50s_ values of 0.63, 0.67, and 0.00137 µM at 24, 48, and 72 h, respectively. Under similar conditions, SZ and PY, considered as the gold standard drugs for the treatment of toxoplasmosis, had IC_50s_ values of 1.29, 1.55, and 0.95 and 3.19, 3.52, and 2.42 µM, respectively. The rapid dose-dependent inhibition of *T. gondii* tachyzoites by DHQ compared to the standard drugs (SZ and PY) indicates that DHQ has high selective parasiticidal effects against tachyzoite proliferation. Remarkably, DHQ had an excellent selectivity index (SI) of 149- and 357-fold compared to 24- and 143-fold for PY and SZ, respectively, using fibroblast cells. In addition, DHQ disrupted *T. gondii* tachyzoite mitochondrial membrane potential and adenosine triphosphate (ATP) production and elicited high reactive oxygen species (ROS) generation. Taking all these findings together, DHQ promises to be an effective and safe lead for the treatment of toxoplasmosis.

## Introduction

*Toxoplasma gondii* is an obligate intracellular parasite that causes toxoplasmosis. Globally, between 30% and 50% of humans (Pappas et al., 2009; Flegr et al., 2014) and 35% and 76% of wild and domestic felids are infected with *T. gondii* (Montazeri et al., 2020). According to the Centers for Disease Control and Prevention (CDC), *T. gondii* is one of the leading causes of foodborne-related illness in the United States (CDC, 2021). Additionally, it has been reported that *T. gondii* infections negatively impact the US economy (Ben-Harari and Connolly, 2019).

Importantly, studies have shown a positive correlation in molecular signatures between suicide rate, schizophrenia, Alzheimer’s and Parkinson’s diseases, and *T. gondii* infections (Kamerkar and Davis, 2012; Gajewski et al., 2013; Ngô et al., 2017; Mara, 2018; Abo-Al-Ela, 2019).

Currently, the combination of pyrimethamine (PY) and sulfadiazine (SZ) is the first line of therapy against *T. gondii* infections (Angel et al., 2020; Secrieru et al., 2020; Shammaa et al., 2021). For congenital toxoplasmosis, spiramycin is used as a prophylactic treatment (Secrieru et al., 2020). For cerebral toxoplasmosis, co-trimoxazole or atovaquone are the most effective drugs recommended for treatment; for *T. gondii* retinocoroiditis, clindamycin, co-trimoxazole, atovaquone, and prednisone are the most effective drugs (Secrieru et al., 2020). These drugs have serious adverse side effects, impeding their use for all cases of toxoplasmosis (Shiojiri et al., 2019; Angel et al., 2020; Montazeri et al., 2020; Secrieru et al., 2020; Shammaa et al., 2021; Wang et al., 2021). Thus, there is an urgent need to discover new drugs that inhibit *T. gondii* tachyzoites, the replicative form of the parasite, because of its ability to spread to all organs and its conversion to bradyzoites within tissue cysts (Black and Boothroyd, 2000). Immunosuppression, like in AIDS patients, may result in reactivation of the quiescent tissue cyst causing life-threatening cerebral toxoplasmosis (Luft and Remington, 1992; Luft et al., 1993; Black and Boothroyd, 2000).

Antimalarial compounds such as quinine-based compounds—quinine (Q), dihydroquinine (DHQ), chloroquine (CQ), quinidine (QD), and dihydroquinidine—have been reported to be effective against the human and rodent malaria parasites *Plasmodium falciparum* and *Plasmodium berghei* (Polet and Barr, 1968; Brossi et al., 1971; Brossi et al., 1973; Nontprasert et al., 1996; Pukrittayakamee et al., 1997; Sanchez et al., 2008; Achan et al., 2011; Gorka et al., 2013; Prachayasittikul et al., 2013). In addition, quinine has been reported to inhibit *T. gondii*, *Besnoitia jellisoni*, and *Sarcocystis* sp. host cell invasion (Fayer et al., 1972). Interestingly quinine sulfate, a derivative of quinine, exhibited a strong inhibition of skin pathogenic bacteria invasion and internalization of host cells (Wolf et al., 2002; Wolf et al., 2006). Interestingly, DHQ is an impurity often associated with quinine drugs formulations constituting up to 10% and has potent activity against *Plasmodium* species (Nontprasert et al., 1996). Little is known about the effect of the DHQ effect on *T. gondii*, which belongs to the same phylum Apicomplexan as *Plasmodium* spp. and *B. jellisoni*. Further, the DHQ mechanism of action against *T. gondii* growth is not known. In this study, we report DHQ inhibition of *T. gondii* growth, invasion, disruption of mitochondrial membrane, adenosine triphosphate (ATP) production, and induction of elevated reactive oxygen species (ROS) *in vitro.*

## Materials and Methods

### Parasites and Host Cell Cultures

hTERT cells (obtained from Silvia Moreno’s Laboratory at the University of Georgia, Athens, GA) were maintained using Dulbecco’s modified Eagle’s medium (DMEM) media supplemented with 1% of penicillin–streptomycin (PS) and Amphotericin B and 10% fetal bovine serum (FBS) (Thermo Fisher Scientific Inc., Waltham, MA, USA). Cells were maintained at 37°C with 5% CO_2_. The RH-W and RH-RFP (Type I) strains (a gift from Prof. Silvia Moreno) were maintained in DMEM containing 1% PS and 10% FBS or 10% fetal calf serum (FCS) obtained from Sigma-Aldrich (St. Louis, MO, USA).

### Inhibition of Intracellular RH-RFP Tachyzoite Growth

To test the anti-*T. gondii* inhibition of DHQ, an RH-RFP (Type I) *T. gondii* parasite genetically engineered to express a red fluorescent protein was used (Szajnman et al., 2017). First, hTERT cells (6.0 × 10^4^ cells/ml) were counted and seeded into 96-well plates/100 µl and incubated at 37°C supplemented with 5% CO_2_ to grow for 24 h according to previous publications (Li et al., 2017; Szajnman et al., 2017). At 24 h, wells were washed with 1× phosphate-buffered saline (PBS) twice, and 100 µl of RH-RFP (6.3 × 10^4^ cells/well) tachyzoites was added to the same 96-well plates and allowed to infect host cells for 3 h at 37°C with 5% CO_2_ and 95% atmospheric air (Leesombun et al., 2016; Zhang et al., 2019a; Zhang et al., 2019b; Salin et al., 2020; Contreras et al., 2021; Wang et al., 2021). At 3 h, plates were removed from the incubator, and extracellular parasites and the medium were washed off twice with 1× PBS followed by the addition of drugs serially diluted using procedures published by (Salin et al., 2020; Nurkanto et al., 2021; Costa et al., 2021). A volume of 100 µl of DHQ (0, 3.13, 6.25, 12.5, 25, and 50 µM), positive controls (SZ and PY with concentrations of 0, 3.13, 6.25, 12.5, 25, and 50 µM), and negative control (culture media) were added to the designated wells. Plates were read at 24, 48, and 72 h using Tecan 200 F infinite microplate reader set at excitation of 560 nm and emission of 630 nm; the gain was set to 100% optimal with 25 flashes. Plates were read from the top. The percent of parasites growth was calculated using the following formula: (Average control fluorescence wells minus treated fluorescence wells/Average control fluorescence) × 100. From these calculations, the percent inhibition of parasites growth was determined by subtracting parasite growth percent by drugs from 100% growth by the controls (clear culture medium with parasites without drugs). The IC_50s_ were calculated for 24, 48, and 72 h using a non-linear regression approach, where the concentrations were transformed to log_10_ and plotted against the percent inhibition of *T. gondii* growth using GraphPad Prism software version 9.0.

### Effect of Dihydroquinine Extracellular Pretreatment on *Toxoplasma gondii* Invasion of hTERT Cells

To evaluate whether DHQ could prevent tachyzoite invasion of host cells, hTERT cells (2 × 10^5^ cells/2 ml) were seeded onto glass coverslips using 6-well plates and allowed to grow to 80% confluency. To perform the invasion assay, compounds DHQ (6.25 and 50 µM), SZ (50 µM), PY (6.25 µM), and PY/SZ (1.56/50 µM) of 100 µl were added to freshly egressed tachyzoites (1.70 × 10^4^ parasites/100 µl) that were purified with Whatman filter of size 3 µm, kept in 1.5-ml Eppendorf tubes, and then incubated for 30 min at 37°C with 5% CO_2_ with the occasional manual shake. Next, the pretreated extracellular parasites were mixed thoroughly, 100 µl of the mixture was added into 6-well plates containing coverslips with confluent hTERT cells supplemented with 2 ml of fresh medium and allowed to infect the host cells for 1 h, followed by washing away the extracellular parasites using sterilized 1× PBS, performed 3 times, fixed with 100% ethanol, and incubated overnight at 4°C. The next day, the ethanol was taken out followed by three washes with 1× PBS, then stained with hematoxylin for 10 min, washed with 50% ethanol-H_2_O for 5 min, then stained with Eosin for 5 min, and washed with 50% ethanol-H_2_O (León-Nava et al., 2016; Muñiz-Hernández et al., 2021). Plates containing stained parasites were visualized for parasitophorous vacuole (PV) formation using the Zeiss 100 phase-contrast fluorescence microscope coupled with an Amscope camera. Five fields were randomly captured per coverslip, and the number of distinct PVs was counted and compared with the control (culture medium). The experiments were performed in duplicate and presented as % PV inhibition plus SD. *p <* 0.05 was considered as statistically significant to the control and the different drug treatment groups.

### Measurement of Reactive Oxygen Species in *Toxoplasma gondii*

To verify whether DHQ had any oxidative stress effect on parasites, fresh wild-type RH parasites were harvested by passing them through a 27-gauge needle followed by filtration using a 3-µm filter. RH-W parasites (1.60 × 10^6^ parasites/50 µl/well) were seeded into black 96-well plates and treated with H_2_O_2_ (500 µM) as a positive control (Ma et al., 2021), ROS buffer was used as a negative control, and DHQ at different concentrations (6.25 and 50 µM) was used as an experimental drug and incubated for 30 min at 37°C with 5% CO_2_. After the 30-min incubation, 50 µl of H2DCFDA was added, covered with aluminum foil, and incubated for 45 min at 37°C in the dark according to the procedure reported by Charvat and Arrizabalaga (2016). Fluorescence intensities of the wells were measured at excitation 485 and emission 535 nm using a Tecan 200F infinite microplate reader.

### Dihydroquinine Effect on *Toxoplasma gondii* Mitochondrial Membrane Potential (ΔΨm)

For mitochondrial membrane potential measurements, the cationic probe JC-1 was used as previously described by some studies (Charvat and Arrizabalaga, 2016; Syn et al., 2017; Adeyemi et al., 2017; Zhang et al., 2019a; Zhang et al., 2019b). Briefly, freshly purified tachyzoites of 10^5^/50 µl were seeded into black 96-well plates with a clear bottom (Costar, Corning Inc., New York, NY, USA) without host cells followed by the addition of DHQ at concentrations of 6.25, 12.5, and 50 µM of H_2_O_2_ (500 µM) as a positive control and the reaction buffer as negative control and incubated for 8 h at 37°C with 5% CO_2_ according to the manufacturer’s procedure and procedures used in *T. gondii* by some studies (Charvat and Arrizabalaga, 2016; Zhang et al., 2019a; Zhang et al., 2019b). After the 8-h incubation period (Zhang et al., 2019a; Zhang et al., 2019b), 5 µl of JC-1 was added to both controls and drug-treated wells. The reaction plates were covered with aluminum foil and incubated for 45 min followed by centrifugation at 12°C for 2,000 rpm for 5 min (Charvat and Arrizabalaga, 2016; Zhang et al., 2019a; Zhang et al., 2019b). The supernatant was carefully removed, and 100 µl of the assay buffer (AB) was added to each well and centrifuged again under the same condition. The process was repeated a second time, the wells were refilled with 100 µl of AB, and the parasites’ fluorescence was read at 535/630 nm for the JC-1, J-aggregates and at 485/535 nm for the J-monomers using Tecan 200F infinite microplate reader. The ratio of fluorescence values of J-aggregates to fluorescence values of monomers was calculated using the following formula: RFU J-aggregates/RFU J-Monomers. Images were taken at each treated dose and controls using an EVOS FL fluorescence microscope. For the plate reader data, graphs and statistical differences were obtained using Graph Pad Prism software 9.0.2. A one-way ANOVA was carried out using the Holm–Sidâk multiple-comparisons test with *p <* 0.05. Means with *p <* 0.05 were considered as statistically significant relative to controls and the different treated doses/compounds.

### Dihydroquinine Effect on *Toxoplasma gondii* Adenosine Triphosphate Production

To further validate our mitochondrial assay, a modified protocol by Zhang et al. (2019a and 2019b) was used. Fresh extracellular RH-RFP parasites (8.05 × 10^4^ per group) were incubated in a complete medium containing DHQ at concentrations of 6.25, 12.5, and 50 µM or without the drug (medium only with parasites as negative control) and 500 µM of H_2_O_2_ as a negative control. After 8 h of incubation, at standard culture condition of 5% CO_2_ at 37°C, the ATP detection assay kit luminescence (700410; Cayman Chemical, MI, USA) was used to measure the luminescence of samples treated with controls. Experimental drug-treated (DHQ), positive control (H_2_O_2_), and negative control (medium with parasites without drugs) were washed twice with PBS, and the pellet was lysed on ice with 100 µl of lysis buffer. Next, 10 µl of the parasite’s lysates were added to 100 µl of ATP detection working solution in each opaque microplate well (Corning Inc., NY, USA). Plates were incubated for 20 min at room temperature according to the ATP detection kit protocol, the cover of the plates was removed, and the luminescence was measured using BioTek Cytation/3 imaging reader with software Gen 5 3.1 (MA, USA). The effect of treatments on the *T. gondii* ATP luminescence was compared with ATP luminescence of untreated *T. gondii* population (medium with parasites without experimental drugs or negative controls) and expressed as percentages of these control ATP values. Experiments were conducted in three independent experiments and presented as means plus SD of triplicate trials.

### Dihydroquinine Effect on hTERT Mitochondrial Membrane Potential (ΔΨm)

hTERT cells were seeded onto a black 96-well plate at the cell density used for the extracellular parasites’ mitochondrial membrane potential testing as indicated above. At 80% confluence, the medium was removed and washed 3 times with 1× PBS. Drugs were added at similar concentrations used for the parasite mitochondrial membrane potential assay and incubated for 8 h. At 8 h, the JC-1 dye was added, wrapped with aluminum foil, and incubated for 45 min at standard culture conditions. At 45 min, plates were removed from the incubator and centrifuged for 5 min at 12°C for 2,000 rpm. The supernatant was carefully removed, replaced with 100 µl of AB, and centrifuged. The supernatant was discarded one more time and replaced with 100 µl AB and centrifuged, and the supernatant containing JC-1 was carefully removed. Next, 100 µl of the AB was added to the wells, and their fluorescence intensities were recorded at 535/630 nm for the JC-1, J-aggregate form and 485/535 nm for the J-monomers using Tecan 200F infinite microplate reader. The ratio of fluorescence values was determined as stated above. Photomicrographs were taken using EVOS FL fluorescence microscopy. Graphs and statistical differences were obtained using Graph Pad Prism software 9.0.2. *p* ≤ 0.05 was considered as statistically significant relative to controls and different treated doses.

### Cytotoxicity of Dihydroquinine, Sulfadiazine, Pyrimethamine, and Sulfadiazine/Pyrimethamine on Host Cells

#### CyQuant^®^ NF Cell Proliferation Determination Using hTERT Cells

Exactly 6.0 × 10^4^ cells/well were grown on black 96-well plates at 100 µl for 24 h. Next, at 24 h, medium with dead cells was carefully removed, and the remaining cells were washed three times with 1× PBS and then incubated in 100 µl of drugs at concentrations of 0, 3.13, 6.25, 12.5, 25, and 50 µM for DHQ, SZ, PY, and PY/SZ. Standard culture medium with no phenol red was used as a negative control. At 72 h, NF dye solution was added to wells, covered with aluminum foil, and incubated in standard culture condition for 1 h, and the fluorescence intensity read was at excitation of 485 nm and emission of 535 nm. The percent viability was calculated by subtracting the blank (medium with cells without drugs) from the treated wells. Cell viability was determined using the following formula: (treated/control) × 100.

### Statistical Analysis

The IC_50s_ and CC_50s_ were determined using Graph Pad Prism 9.0.2 software. All experiments were performed in duplicate, with each experiment performed in triplicate wells for statistical analysis purposes. A one-way ANOVA was carried out using the Holm–Sidâk multiple-comparisons test on treated samples relative to the controls or in other cases different doses/treatments. A statistically significant difference was reported, where a value of *p <* 0.05. Also, the selectivity index (SI) was used to determine if the compounds of interest were inhibiting the tachyzoite growth but not toxic at similar concentrations to host cells.

## Results

### Dihydroquinine Inhibited *Toxoplasma gondii* RH-RFP Tachyzoite Growth

**Figure 1:**
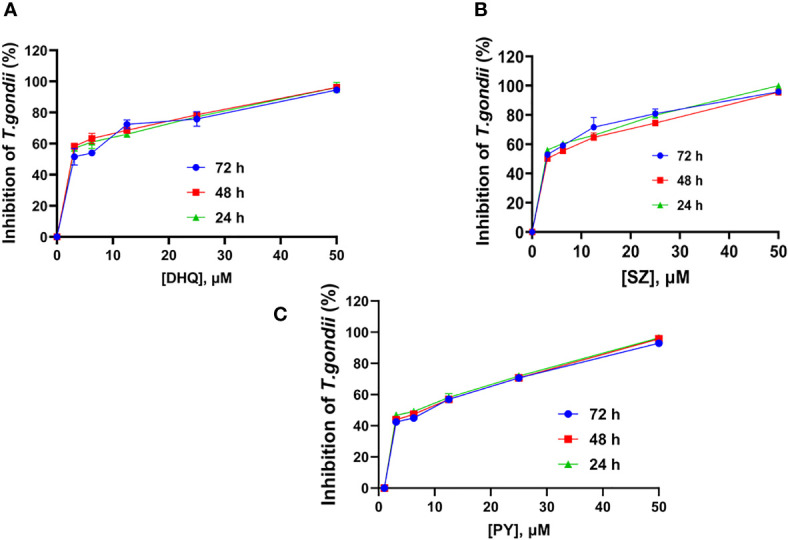
Mean time-dose Kinetic inhibition curve of **(A)** DHQ, **(B)** SZ, and **(C)** PY against *T. gondii* tachyzoites growth in vitro at 24, 48 and 72 hours interaction.

We assessed the anti-*T. gondii* activity of DHQ as the compound of interest and compared its effect with the standard drugs SZ and PY *in vitro*. DHQ inhibited tachyzoite growth in a dose- and time-dependent manner (**Table 1**). The 50% minimum inhibitory concentration (IC_50s_) values after 24, 48, and 72 h of interaction between DHQ and parasites were 0.63, 0.68, and 0.0014 µM, respectively. The IC_50s_ for SZ and PY after the same times of interaction were 1.29, 1.55, and 0.95 µM for SZ and 3.19, 3.52, and 2.42 for PY. Surprisingly, at 72 h, DHQ showed an IC_50_ value in the sub-nanomolar level as compared to the IC_50_ obtained at 24 and 48 h. Similarly, SZ and PY were more effective at 72 h than 24 and 48 h. Also, of interest was the fact that at concentrations between 6.25 and 50 µM, there was maximum death of *T. gondii* tachyzoites ranging from 70% to 100% with DHQ, SZ, and PY at different time points selected for the study (**Figures 1A–C**).

**Table 1:** Mean kinetic IC_50s_ (µM) of DHQ, SZ, and PY on intracellular *Toxoplasma gondii* tachyzoite growth inhibition.

Compounds	IC_50_ @ 24 h	IC_50_ @ 48 h	IC_50_ @ 72 h
DHQ	0.63 ± 0.0024	0.67± 0.27	0.0014 ± 0.00
SZ	1.28 ± 0.33	1.55 ± 0.048	0.95 ± 0.35
PY	3.18 ± 0.37	3.52 ± 0.27	2.42 ± 0.34

Data are reported as means IC_50s_ plus SD of three independent experiments performed in triplicate each. hTERT cells (6.0 × 10^4^ cells/ml) were grown for 24 h, and non-adherent cells were washed away. Next, 100 µl of RH-RFP (6.3 × 10^4^ cells/well) tachyzoites was added to wells and incubated for 3 h, followed by 1× phosphate-buffered saline (PBS) wash to remove all the extracellular parasites, leaving only intracellular parasites. Wells were treated with 100 µl volume of compounds (DHQ, SZ, and PY). Parasites fluorescence was recorded for 24, 48, and 72 h, and the 50% minimum inhibitory concentrations of the parasites’ growth were determined. Prior to the IC_50s_ calculations, the concentrations were converted to log and plotted against percent inhibition using a GraphPad Prism. DHQ (dihydroquinine; current compound studied), SZ (sulfadiazine), and PY (pyrimethamine) are the primary known drugs used in combination for the treatment of T. gondii infection. IC_50_ is the minimum concentration that will inhibit 50% of T. gondii parasites’ growth.

### Dihydroquinine Reduces Extracellular Tachyzoite Invasion of Host Cells

**Figure 2:**
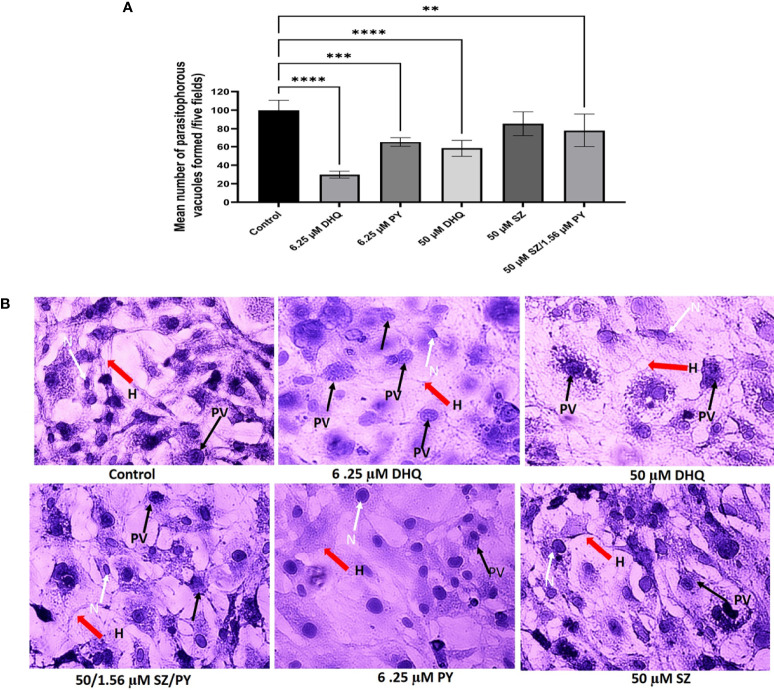
**(A)** DHQ disrupts extracellular *T. gondii* tachyzoites ability to invade human foreskin fibroblast (hTERT) cells (2 x 10^5^/2 ml). To evaluate the effect of DHQ on *T. gondii* tachyzoites invasion of host cells extracellular parasites (1.70 x 10^4^ parasites/100 μL) were treated with DHQ at concentrations of 6.25, and 50 μM. PY and SZ were used as standard controls at concentrations (6.25 and 50 μM), respectively. 50μM/1.56 μM of SZ/PY was used as a positive control for a standard combination treatment. Data are means ± standard errors of duplicate experiments performed in triplicate. * represent a significance difference between 50 μM/1.56mM of SZ/PY and the control (*p* < 0.01). ** indicates a significant difference between 6.25 μM relative to the control (p<0.01); *** indicates a significant difference between 6.25 μM and 6.25 μM PY (p < 0.01); **** indicates a significant difference between 6.25 μM DHQ and 50 μM/1.56 μM of SZ/PY, and control 50 μM DHQ and control (p < 0.01). **(B)** Representative images of different concentrations of DHQ (6.25 and 50 μM), SZ (50 μM), PY (6.25 μM) and SZ/PY (50/1.56 μM) combination treatment on extracellular T. gondii tachyzoites for 30 minutes and release into fresh hTERT cells to invade for one hour. Plates were washed with 1X PBS thrices to remove uninvaded parasites and stained with hematoxylin-eosin followed by visualization of PVs formed using Zeiss 100 phase-contrast microscope integrated with Amscope camera. The red arrows resepresent host cells (H), and the black arrows represent tachyzoites in parasitophorous vacuoles (PVs), and white arrows and N represent the nucleus of the cells.

We observed that DHQ affected the capacity of tachyzoites to infect hTERT cells in a dose-dependent manner within a short time of 30 min of extracellular exposure to infection of host cells (**Figure 2A**). Also, we observed that there were fewer PVs formed with a low concentration of DHQ = 6.25 µM and at a high concentration of 50 µM, confirming our inhibition assay that showed that DHQ reduces tachyzoite replication *in vitro* (**Figure 2B**). The different doses of DHQ used were significantly different relative to those of other treatments (SZ, PY, and SZ/PY) and control groups (*p* < 0.05). A similar trend was observed with PY, SZ, and PY combination concentrations tested. However, treatments with 50 µM of SZ were not significantly different from the control *p* > 0.05 (**Figure 2A**).

### Dihydroquinine Induces Reactive Oxygen Species Generation in *Toxoplasma gondii*

**Figure 3:**
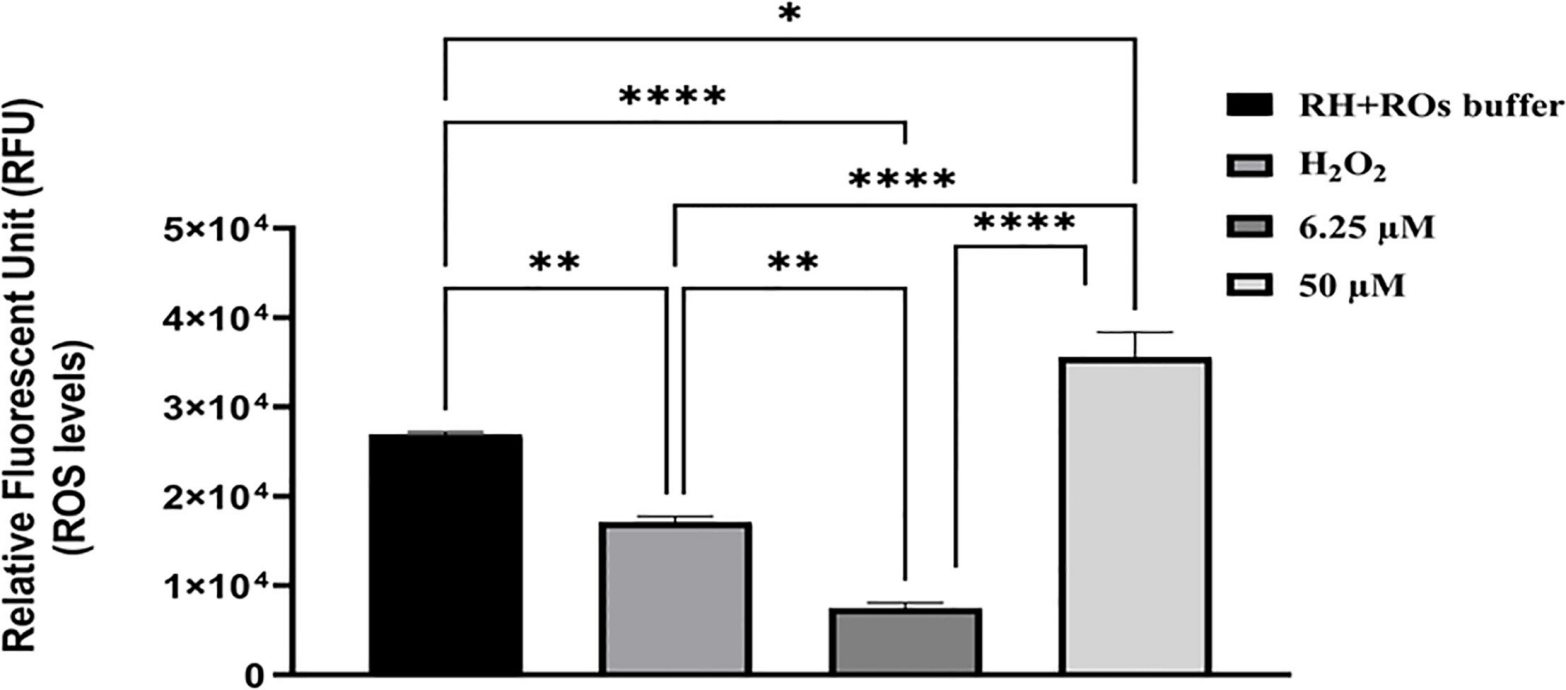
DHQ caused elevated reactive oxygen species (ROS), in *T. gondii* (R-W strain) tachyzoites. To evaluate the effect of DHQ on *T. gondii* (RH-Wild type) tachyzoites reactive oxygen species (ROS) generation, extracellular parasites (1.60 x 10^6^ parasites/50 μL) were purified by syringe and filtered using 3 μm filter. Next same parasites numbers were treated with DHQ concentrations of 6.25, 12.5 and 50 μM. Hydrogen peroxide (H_2_O_2_) at 500 μM, and RH-ROs buffer were used as positive controls. Plates were incubated for 30 minutes followed by the addition of ROS dye and incubated for 45 minutes. Fluorescence were measured at 485 nm excitation and 563 nm emission. Data are presented as means plus standard errors of three different experiments performed in triplicate and analyzed using one-way ANOVA with comparison of means using Holm-Šídák multiple comparison with *p* < 0.05. * represent a significance difference between 50 μM and RH-ROs buffer (negative control) (*p* < 0.004). ** indicates a significant difference between H_2_O_2_ and 6.25 μM (*p* < 0.004); **** indicates a significant difference between the following groups; H_2_O_2_ and 50 μM (*p* < 0.0001), 6.25 μM and 50 μM (*p* < 0.0001), 6.25 μM and RH-ROs buffer group (p < 0.0001).

DHQ is a synthetic organic compound produced during the natural alkaloid (quinine) synthesis as commercial waste with antimalarial properties (Polet and Barr, 1968; Brossi et al., 1971; Brossi et al., 1973; Nontprasert et al., 1996; Pukrittayakamee et al., 1997; Sanchez et al., 2008; Achan et al., 2011; Gorka et al., 2013). Based on the above evidence of DHQ coupled with the remarkable inhibition and anti-invasion data obtained, we tested DHQ against extracellular freshly purified parasites and found that DHQ at 50 µM induced a little higher ROS level in *T. gondii* tachyzoites *in vitro* in a concentration-dependent manner (**Figure 3**). Interestingly, treatment at 6.25 µM of DHQ versus 50 µM of DHQ inhibited parasite invasion significantly (*p* < 0.01). A similar trend was found with H_2_O_2_ (positive control) versus 50 µM of DHQ, and ROS buffer (negative control) versus 6.25 µM of DHQ treatment with (*p* < 0.01). We also discovered that the total ROS released from tachyzoites as a result of being treated with H_2_O_2_ (500 µM) as positive control versus 6.25 µM of DHQ or ROS buffer treatment versus H_2_O_2_, or ROS buffer treatment versus 50 µM of DHQ were significantly different with *p* < 0.02.

### Dihydroquinine Causes Mitochondrial Membrane Damage in *Toxoplasma gondii* But Not of the Host Cells

**Figure 4:**
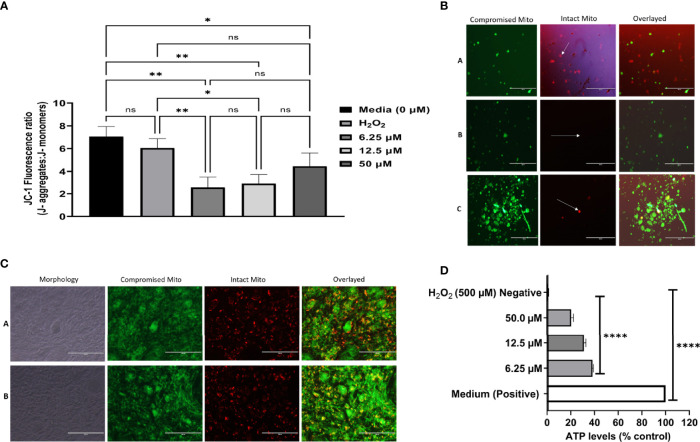
**(A)** DHQ disrupts mitochondrial membrane potential in T. gondii tachyzoites. To evaluate the effect of DHQ in T. gondii tachyzoites mitochondria, extracellular parasites (10^5^/50 μL) were suspended with DHQ concentrations ranged from 6.25, 12.5 and 50 μM. Hydrogen peroxide (H_2_O_2_) was used as a positive control. Data are presented as means plus standard errors of three different experiments performed in triplicate. * represent a significance difference between 50 μM relative to the control (p < 0.038). ** indicated a significant difference between 6.25, 12.5, μM DHQ relative to the control (0 μM) with (p < 0.002); Also, there was a significant difference between H_2_O_2_ versus 6.25 (p < 0.0079), H_2_O_2_ versus 12.5 μM DHQ (p = 0.014); ns, indicates no significant difference between the controls and the treated. **(B)** Morphological changes of T. gondii tachyzoites exposure to DHQ for 8 hours. Briefly, 10^5^ RH-wild type of T. gondii was purified with syringe and filtered with 3 μm filter. Next 50 μL of the parasites were seeded into black 96 well plates and the reactions were allowed for 8 hours at 37 °C at 5% CO_2_. After 5 μl of JC-1dye was added and covered with foil and incubated fir 45 minutes followed by sequential centrifugation, and fluorescence taken using EVOS FL Fluorescent microscope. Buffer was used as control **(A)**, 12.5 μM DHQ **(B)** and 50 μM DHQ **(C)**. Data are a representative of three independent experiments conducted in triplicate. The green represent parasites mitochondrial membrane potential disrupted by DHQ and the while arrow pointing to the red spots tachyzoites with no disruption of mitochondria membrane [see **(A)**]. The more JS-1 accumulation in the mitochondrial the greener the tachyzoites will appear. **(B, C)** were remarkable different compared with the A which was a negative control. Bar scale: 200 μm. **(C)** DHQ does not disrupts mitochondrial membrane potential in hTERT cells. To evaluate the effect of DHQ on host cells mitochondria disruption, intracellular hTERT cells (10^6^) were treated with DHQ concentration of 50 μM. Hydrogen peroxide (H_2_O_2_) was used as a positive control at 500 μM. Data are reported as representative from three independent experiments determined in triplicate. ROS Buffer was used as negative control **(A)** and 50 μM as drug **(B)**. A1 and B are light microscope images showing how the hTERT cells morphological looks after the control and drug treatment for ROS generations using the same exposure period tested with the parasites. Bar scale: 200 μm. **(D)** DHQ reduced ATP levels in *T. gondii* tachyzoites. To evaluate the effect of DHQ on *T. gondii* tachyzoites ATP levels in, extracellular parasites (104/50 μL) were suspended with DHQ concentrations ranged from 6.25, 12.5 and 50 μM. Hydrogen peroxide (H_2_O_2_) was used as a negative control. Data are presented as means plus standard deviation of three different experiments performed in triplicate. * represent a significance difference between 50 μM relative to the control (*p* < 0.038). ** indicated a significant difference between 6.25, 12.5 μM DHQ relative to the control (0 μM) with (*p* < 0.002); Also, there was a significant difference between H2O2 versus 6.25 (*p* < 0.0079), H_2_O_2_ versus 12.5 μM DHQ (*p* < 0.014).

It has been reported in the literature that a high level of ROS could affect lipids, proteins, DNA, and mitochondrial energy (Lill et al., 2014; Zhao et al., 2015). Based on our previous data of the ROS study, we wanted to decipher whether DHQ affected the *T. gondii* mitochondrial membrane potential. Here, we used the cationic JC-1 dye to decipher the effect of DHQ on *T. gondii* tachyzoite mitochondrial membrane potential (ΔΨm). This dye has been used to decipher mitochondrial health in parasites (Charvat and Arrizabalaga, 2016; Zhang et al., 2019a; Zhang et al., 2019b). Surprisingly, we observed that DHQ induced mitochondrial membrane damage in *T. gondii* tachyzoites in a concentration-dependent manner but not on host cells’ mitochondrial membrane potential at concentrations of 6.25, 12.5, and 50 µM (**Figures 4A, B**). It was observed that treatment with H_2_O_2_ as a positive control compared with 6.25 and 12.5 µM exhibited a statistically significant difference (*p <* 0.01 and *p* = 0.01, respectively). Comparison of the negative control (Medium) with 6.25 or 12.5 or 50 µM of DHQ treatment on extracellular parasites showed a significant difference (*p <* 0.01, *p <* 0.01, and *p* = 0.04, respectively). **Figure 4C** shows a representative image of the DHQ effect on the mitochondrial membrane potential of hTERT cells (host cells). Remarkably, our studies showed that DHQ does not affect host cells’ mitochondrial membrane potential as shown in **Figure 4C**. A similar result was reported for other mitochondrial membrane potential inhibitors of *T. gondii* (Zhang et al., 2019a; Zhang et al., 2019b; McPhillie et al., 2020). Thus, the DHQ mechanism of action was solely on the parasite mitochondria and not due to the influence of the hTERT cells.

### Dihydroquinine Effect on the Adenosine Triphosphate Level in *Toxoplasma gondii*

Different doses of DHQ were observed to exert a reduction in *T. gondii* ATP production *in vitro* (**Figure 4D**). Generally, we observed significant statistical differences between the positive (H_2_O_2_) control and 6.25, 12.5, or 50 µM (*p <* 0.01). Similarly, we observed significant statistical differences between the ATP levels of this DHQ with concentrations of 6.25, 12.5, and 50 µM used (*p <* 0.01). Lastly, the negative controls were significantly different from both experimental drug tested and positive control with *p <* 0.01.

### Dihydroquinine Effect on Host Cell Viability

#### hTERT Cells

To validate that our inhibition results on parasites were not due to cytopathic effects on the hTERT cells, we tested all compounds’ (DHQ, SZ, and PY) cytotoxicity using serially diluted concentrations of 0, 3.13, 6.25, 12.5, 25, and 50 µM using the same protocol for parasite inhibition assay, with 72-h time exposure to all compounds. We found that treatment with 0.0 to 50 µM of the DHQ compound had no effect on host cell viability (**Supplementary Figure 5A**).

Also, to prove that DHQ does not alter the morphology of the host cells due to toxicity, we have presented representative images of host cells at selected concentrations (**Supplementary Figure 5B**). The CC_50s_ were determined to be 209.1, 135.4, and 57.4 µM for DHQ, SZ, and PY, respectively (**Table 2**).

**Table 2:** Mean CC_50s_ (µM) of DHQ, SZ, and PY on hTERT cells.

Compounds	CC_50_ @ 72 h (LL ± UL)	SI
DHQ	209.1 (121.7 ± 501.8)	149,357.14
SZ	135.4 (76.9 ± 313.7)	142.53
PY	57.34 (42.90 ± 78.79)	23.69

Data are reported as means IC_50s_ plus SD of three independent experiments performed in triplicate each. hTERT cells (6.0 × 10^4^ cells/ml) were grown for 24 h, and non-adherent cells were washed away. Microplate wells were treated with 100-µl volume of compounds (DHQ, SZ, and PY) as described in the anti-parasitic assay for uniformity purposes. The 50% minimum cytotoxic concentrations of the hTERT cells were determined. Prior to the CC_50s_ calculations, the concentrations were converted to log and plotted against the percent viability of host cells using a GraphPad Prism. DHQ (dihydroquinine; current compound studied) SZ (sulfadiazine), and PY (pyrimethamine) are the primary known drugs used in combination for the treatment of Toxoplasma gondii infection. IC_50_ is the minimum concentration that will inhibit 50% of T. gondii parasites’ growth. CC_50_ (minimal cytotoxic concentration that will kill 50% of host cells) is presented as triplicate determination (n = 3). SI (selectivity index) is determined by (CC_50@72h_/IC_50@72h_) interaction of compounds host cells/parasites. (LL ± UL), where LL is lower limit and UP is upper limit.

### Dihydroquinine Selectivity Index

Quinine-based drugs though are still useful in the treatment of malaria and other diseases; their toxicity and other side effects have limited their acceptance in certain countries as the first line of treatment. In this study, we determined the SI of DHQ, which is a derivative of the quinine drug, on the host cell to be 149,357 for hTERT cells. In the standard controls, SZ and PY had their SI values to be 142.53 and 23.69, respectively, for human foreskin fibroblast (hTERT) cells challenged for 72 h *in vitro* (**Table 2**).

## Discussion

Toxoplasmosis continues to be a major challenge in both veterinary medicine and public health. Globally, more than 30% of the human population and over 35% of domestic and wild felines are infected with *T. gondii*, respectively (Montoya and Liesenfeld, 2004; Pappas et al., 2009; Robert-Gangneux and Dardé, 2012; Flegr et al., 2014; Montazeri et al., 2020). The consequence of the parasite infection ranges from asymptomatic to life-threatening conditions in both humans and animals (Dubey, 2010; Weiss and Kim, 2011).

Efforts are ongoing to discover novel, safe, and effective vaccines, but none have reached Food and Drug Administration (FDA) approval and commercialization yet, especially for human use (Ducournau et al., 2020). The only option for treating toxoplasmosis in humans has been through antifolate and macrolide drugs (Neville et al., 2015; Shiojiri et al., 2019; Secrieru et al., 2020; Shammaa et al., 2021). However, currently, the use of these drugs is limited geographically, clinically, and socioeconomically (Neville et al., 2015; Shiojiri et al., 2019; Secrieru et al., 2020; Shammaa et al., 2021). Thus, there is an urgent need to discover new compounds or repurposed existing compounds that could be effective, safe, and less costly against parasite infection in humans and even animals. In this study, we investigated DHQ, also known as hydroquinine, for the first time against the RH-RFP (Type I) strain of *T. gondii* growth and the possible mechanism of action *in vitro.* DHQ was found to effectively inhibit parasite growth *in vitro.* This observation agreed with previous studies of this compound on *Plasmodium* species (Nontprasert et al., 1996). Surprisingly, the IC_50s_ values at 24- to 72-h interactions with parasites *in vitro* found that DHQ was highly effective than the current drugs, SZ and PY, as positive standards.

It has been reported that the effect of compounds in intracellular parasites may be influenced by first exerting cellular effects on the host cells’ health, causing parasite stress and eventually death (Munera López et al., 2019). To avoid this, it has been suggested that extracellular parasites be treated with compounds prior to infection of host cells to decipher the compound effects on their infectivity or invasion or replication or PV formation (Zhou et al., 2013). We discovered that pretreatment of tachyzoites with DHQ for 30 min followed by infection of host cells for 1 h significantly affected parasite invasion and replication. Uniquely, the anti-invasion activity was dose and compound dependent. Our finding is supported by earlier studies, using quinine, the DHQ parent compound, which showed inhibition of *T. gondii*, *B. jellisoni*, and *Sarcocystis* spp. host cell penetration (Fayer et al., 1972). Additionally, in prokaryotic cells (i.e., bacteria), quinine derivative (quinine sulfate) has been found to block bacteria invasion and internalization (Wolf et al., 2002; Wolf et al., 2006).

To decipher the mechanism of action of DHQ, we performed several experiments using cellular biochemical assays to tease out whether DHQ could have an effect on the mitochondrial membrane potential and the generation of ROS. Noteworthy, we found that DHQ induces elevated ROS production in *T. gondii* tachyzoites. In the literature, elevated ROS has been shown to contribute to the lethal action of certain antimicrobial, especially the quinolone-based drugs (Zhao et al., 2015). This supports the evidence that compounds that can cause ROS generations to disrupt the mitochondrial detoxifying enzymes such as peroxiredoxin (PRX), superoxide mutase (SOD), and catalase (CAT) act as antioxidants to scavenge the free hydroxyl radicals, superoxide, and hydrogen peroxide (Miller and Britigan, 1997; Vercesi et al., 1998; Odberg-Ferragut et al., 2000; Ding et al., 2000; Kaasch and Joiner, 2000; Son et al., 2001; Kwok et al., 2004).

This conjecture has been reported in *Escherichia coli* and other parasites to cause abnormality of the cellular repair mechanisms (Dwyer et al., 2007; Hong et al., 2019). Also, compounds that cause elevated ROS generations result in secondary damage, which could severely trigger the continuity of ROS accumulations in the intracellular levels, resulting in the disruption of mitochondrial DNA, nucleic acid, lipid, and protein synthesis (Dwyer et al., 2007; Lavine and Arrizabalaga, 2012; Zhao et al., 2015; Rizk et al., 2021). Thus, we believed that this is one of the means by which DHQ exerts its anti-*T. gondii* activity.

Mitochondria is an important organelle that supports cellular respiration, production of ATP, fatty acid degradation, nuclear iron–sulfur protein synthesis, DNA synthesis and repairs, tRNA modification, and protein translation (Antico Arciuch et al., 2012; Lill et al., 2014; Syn et al., 2017). Very importantly, in this study with DHQ, we discovered that it disrupted the mitochondrial membrane potential of tachyzoites that were exposed for 8 h *in vitro* but not host cells. This trend could be attributed to the fact that there is a single mitochondrion in *T. gondii* (Melo et al., 2000; Zhang et al., 2021). Typically, respiration and phosphorylation processes in *T. gondii* are carried out in this single mitochondrion (Vercesi et al., 1998; Bhatia-Kissova and Camougrand, 2010; Narendra and Youle, 2011). Since the mitochondria are crucial for the parasite’s energy requirements for survival, growth, movement, replication, and translational activities (Melo et al., 2000; MaCRae et al., 2012; Zhang et al., 2021), damage to this organelle by DHQ could have resulted in the disruption of tachyzoite invasion of host cells, intracellular growth/replication, and eventually death. DHQ effect on the mitochondrial membrane potential was observed to be dose dependent. For example, at concentrations of 6.25, 12.5, and 50 µM of DHQ interaction with RH-wild type tachyzoites (Type I), we found a significant disruption of the mitochondrial membrane potential. This finding supports previous studies by Lavine and Arrizabalaga (2012), which showed that compounds that cause oxidative stress (e.g., monensin) may disrupt both the cell cycle and mitochondrial function of *T. gondii* tachyzoites by creating autophagy-like cell death in parasites. Furthermore, *T. gondii* mitochondria are at the center of energy generations, and any change in mitochondrial membrane function affects its ATP production and associated functions (Zhang et al., 2019a; Zhang et al., 2019b). Uniquely, our DHQ treatment decreases *T. gondii* ATP levels in a dose-dependent manner and was found to significantly differ from both the positive and negative controls. The current finding that DHQ caused a high reduction of ATP levels in RH-RFP tachyzoites confirmed that DHQ could partly be targeting the oxidative phosphorylation of *T. gondii* parasites. This finding supports several studies that showed that compounds that cause oxidative phosphorylation uncoupling have the tendency to cause disruption of energy metabolism in parasites (Katz, 1986; Frayha et al., 1997; Zhang et al., 2019a; Zhang et al., 2019b). Thus, the reduction of the ATP levels and disruption of mitochondrial membrane potential observed could cause energy shortage and eventually affect parasite invasion, replication, and survival.

Noteworthy, the 50% cytotoxic concentration (CC_50_) of DHQ on hTERT cells was 209.1 µM (209,100 nM), which makes it less toxic than the gold standard drugs PY and SZ, which were found to have high cytotoxic effects on hTERT cells with CC_50s_ of 135.4 µM (135,400 nM) for SZ and 57.34 µM (57,400 nM) for PY. These cytotoxic values for SZ and PY were within the ranges reported in the literature (Rostamkolaie et al., 2019). Comparatively, our result on the CC_50_ of SZ obtained agreed with several studies that have reported the CC_50s_ of SZ in various mammalian cells *in vitro* to be over 1,000 µg/ml (3,995.53 µM) (Leesombun et al., 2016).

Uniquely, the SI of DHQ was over one thousand-fold of what has been reported in SZ and over six thousand-fold more than that of PY. This implies that DHQ is less cytotoxic than both SZ and PY *in vitro.* This finding indicated that the DHQ anti-*T. gondii* activities observed were purely due to their potency against the tachyzoite growth *in vitro* without any cytopathic influence. Several studies have shown that DHQ crosses the blood–brain barrier, has excellent metabolic clearance (Pukrittayakumar et al., 1997; Achan et al., 2011), and thus would have a broad spectrum of inhibition of tachyzoite/bradyzoite growth when successfully developed for the treatment of toxoplasmosis.

## Conclusion

DHQ is highly effective in inhibiting the growth, infectivity of host cells, and mitochondrial membrane potential of tachyzoites *in vitro* in a dose- and time-dependent manner at sub-molar concentrations with negligible effects on human foreskin fibroblast (hTERT) cell viability. DHQ induced high ROS production and induced a decrease of ATP levels in tachyzoites, which explains the disruption of replication, invasion, transcriptional and translational machinery of the parasite, and eventually parasite death due to mitochondrial energy shortage/mitochondrial DNA damage. These findings confirmed that DHQ will be an alternative potent and safe lead compound for the treatment of toxoplasmosis. Our future studies will focus on the medicinal chemistry optimization and evaluation of DHQ and its derivatives *in vivo* to confirm their safety and efficacy for the treatment of cerebral toxoplasmosis based on its ability to cross the blood–brain barrier.

## Data Availability Statement

The original contributions presented in the study are included in the article/**Supplementary Material**. Further inquiries can be directed to the corresponding author.

## Author Contributions

AH, JA, AN, BR, and DA conceived and supported the idea. AH, JA, and DA carried out the experimental works. AH analyzed the data and drafted the first manuscript under the supervision of DA. DA, BR, and AN revised the drafted manuscript. DA and JA performed the ATP depletion assay and revised the manuscript for resubmission. All authors approved the final manuscript.

## Funding

DA had internal funding from the Department of Biological Sciences at Alabama State University (ASU) to perform this work.

## Conflict of Interest

The authors declare that the research was conducted in the absence of any commercial or financial relationships that could be construed as a potential conflict of interest.

## Publisher’s Note

All claims expressed in this article are solely those of the authors and do not necessarily represent those of their affiliated organizations, or those of the publisher, the editors and the reviewers. Any product that may be evaluated in this article, or claim that may be made by its manufacturer, is not guaranteed or endorsed by the publisher.
