# Supplementary figures and images for: Inhibition of Toxoplasma gondii Growth by Dihydroquinine and Its Mechanisms of Action
Source: Front Cell Infect Microbiol. 2022 May 11;12:852889. doi: 10.3389/fcimb.2022.852889 (PMC9131874; doi:10.3389/fcimb.2022.852889)

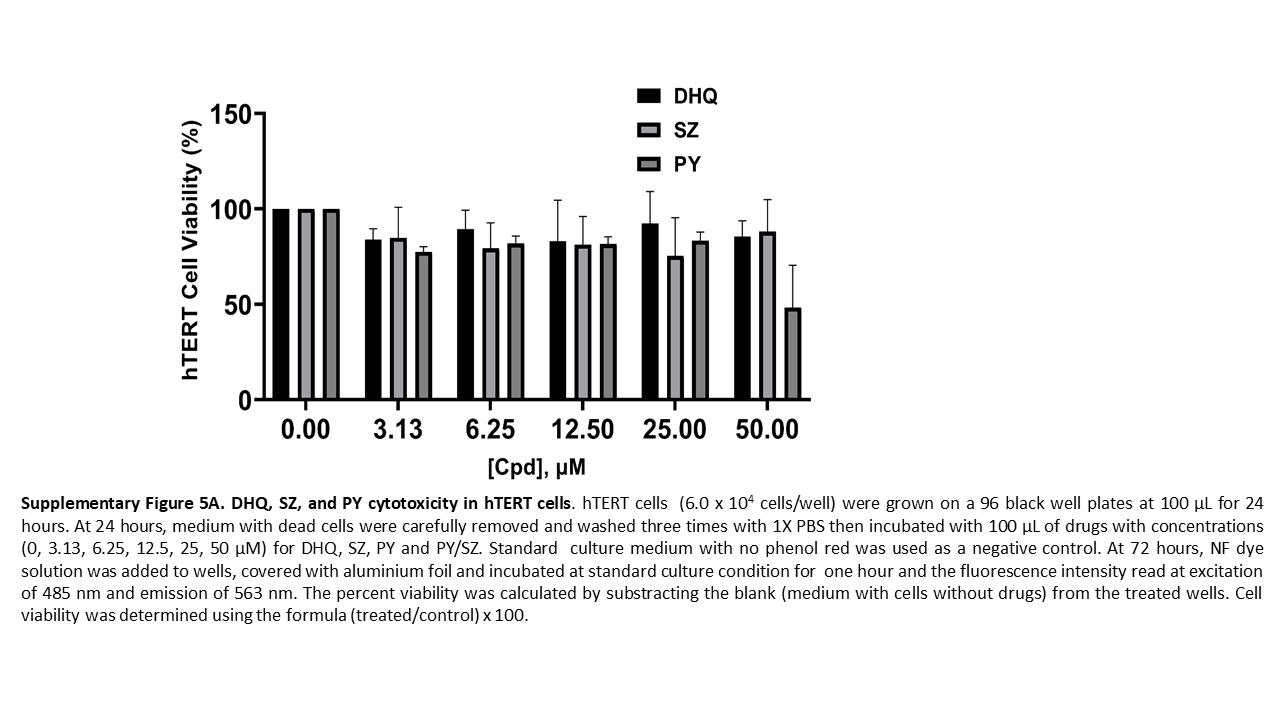

Supplement: Supplementary file 1 [file Image_1.tif]

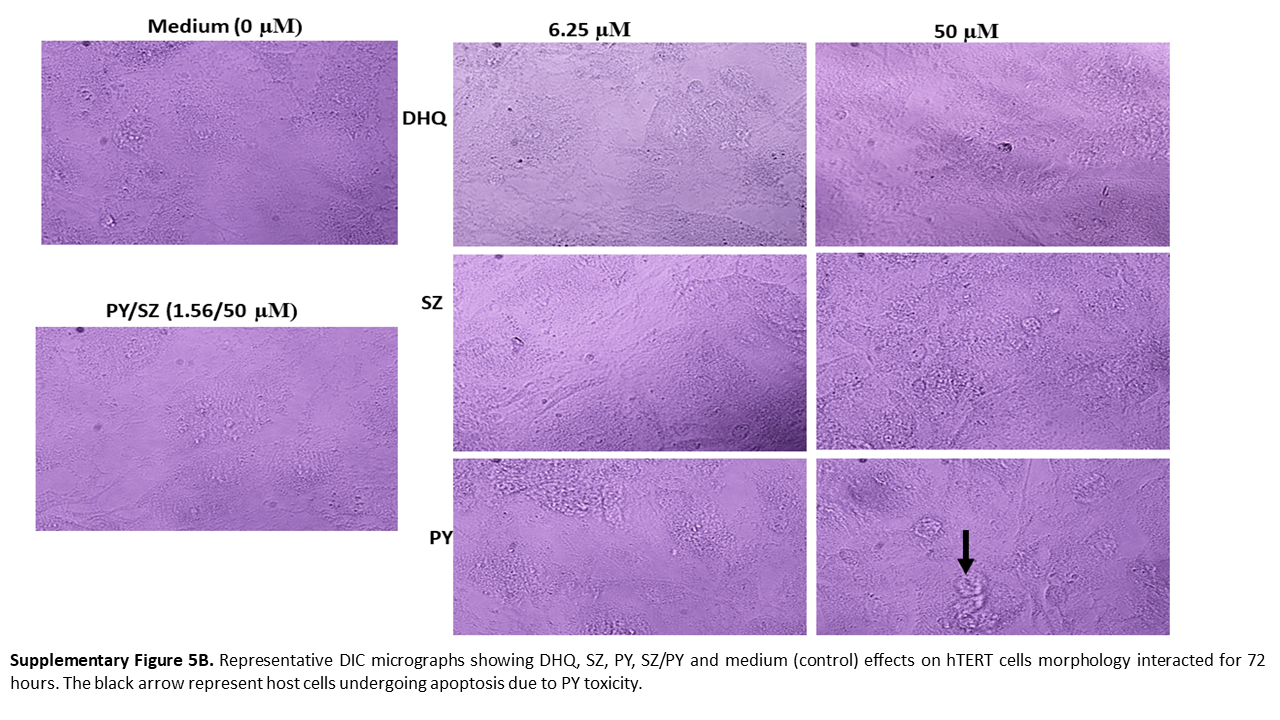

Supplement: Supplementary file 2 [file Image_2.tif]
